# A Dually Flat Embedding of Spacetime

**DOI:** 10.3390/e25040651

**Published:** 2023-04-13

**Authors:** Jan Naudts

**Affiliations:** Departement Fysica, Universiteit Antwerpen, 2610 Antwerpen, Belgium; jan.naudts@uantwerpen.be

**Keywords:** dually flat geometry, spacetime, information geometry, induced matter theory, membrane theory, de Sitter space

## Abstract

A model of spacetime is presented. It has an extension to five dimensions, and in five dimensions the geometry is the dual of the Euclidean geometry w.r.t. an arbitrary positive-definite metric. Dually flat geometries are well-known in the context of information geometry. The present work explores their role in describing the geometry of spacetime. It is shown that the positive-definite metric with its flat 5-d connection can coexist with a pseudometric for which the connection is that of Levi–Civita. The 4-d geodesics are characterized by five conserved quantities, one of which can be chosen freely and is taken equal to zero in the present work. An explicit expression for the parallel transport operators is obtained. It is used to construct a pseudometric for spacetime by choosing an arbitrary possibly degenerate inner product in the tangent space of a reference point, for instance, that of Minkowski. By parallel transport, one obtains a pseudometric for spacetime, the metric connection of which extends to a 5-d connection with vanishing curvature tensor. The de Sitter space is considered as an example.

## 1. Introduction

The embedding of four-dimensional spacetime as a submanifold of a higher-dimensional space is a well-accepted paradigm of theoretical physics. A century ago, Kaluza [1] proposed to embed the four-dimensional spacetime of general relativity in a five-dimensional space in order to unify the theories of electromagnetism and of gravity. A few years later, Klein [2] proposed a way to include quantum effects. In the present work, the quantum reality is neglected, mainly because many approaches to quantization are possible. In addition, the dimension of the embedding space is limited to five. A preliminary version of the present work is found in [3].

The idea that the 4-d curved spacetime becomes flat when extended to higher dimensions is not new. Proponents of the space–time–matter theory [4,5] invoke the theorem of Campbell-Magaard [6] to embed spacetime into a five-dimensional Ricci-flat Riemannian manifold. An alternative interpretation is given by membrane theory, in which spacetime is a hypersurface in a five-dimensional space [7,8]. Higher-dimensional embeddings are in use in the context of string theory and its successor theories [9]. The present work starts from a specific curvature-free connection for the five-dimensional spacetime and explores the curved geometries that can be obtained by selecting a subclass of geodesics and ignoring the motion in the fifth dimension, with the argument that it can be observed only in an indirect manner.

The geometry of four-dimensional spacetime is described locally in good approximation by the Levi–Civita connection of the Minkowski metric, which is a pseudo metric with signature (+,−,−,−) or (−,+,+,+). The metric considered in the present work is positive-definite. This is meaningful because the geometry under study is *not* the one described by the Levi–Civita connection. The main result of the present work is that the positive-definite metric and the pseudo metric can both coexist with a given geometry of the four-dimensional spacetime.

The curvature-free connection of the five-dimensional spacetime chosen in the present work is the dual of the Euclidean connection w.r.t. an arbitrary metric. The dually flat geometry is a corner stone of information geometry [10,11,12]. It derives its relevance from the importance of exponential families, known in Statistical Physics as Boltzmann–Gibbs distributions. The natural parameters of a model belonging to an exponential family are affine parameters of the so-called e-connection, which is one of two flat geometries linked by duality. The hope is that the dual of the Euclidean geometry can play a similar role in the present context.

The coordinates $x^{\mu }$ determine positions in four dimensional spacetime. The additional coordinate is denoted $x^{4}$. Summations in four-dimensional spacetime involve Greek indices μ, ν, ρ, σ, ⋯. In 5-d space Latin capitals *A*, *B*, *C*, ⋯ are used.

The next two sections introduce the dual of the Euclidean connection. The geodesics of this curvature-free connection are characterized by five conserved quantities, denoted $P_{A}$. These are calculated in Section 4. Section 5 shows that fixing $P_{4}$ restores the one-to-one relation between geodesics with a common starting point *x* and common initial velocities ${\dot{x}}^{\mu }$. Starting from Section 6, the choice is made that $P_{4}$, the fifth conserved quantity, vanishes. This allows for an easy calculation of effective connection coefficients and an explicit calculation of the corresponding parallel transport operators. Section 8 uses these operators to construct a pseudometric with the property that the corresponding metric connection coincides with the effective connection introduced before. Section 9 uses the de Sitter space as an example. The final section is a short discussion of the results.

## 2. The Dual Geometry

In five dimensions, a geodesic $t\mapsto x_{t}$ with affine parameter *t* satisfies the equations of motion

(1) $${\ddot{x}}^{A}+\Gamma_{BC}^{A}{\dot{x}}^{B}{\dot{x}}^{C}=0.$$

The coefficients $\Gamma_{BC}^{A}$ are the connection coefficients of the geometry and may still depend on *x* in a smooth way. The 5-d space is treated here as a differentiable manifold. The derivatives ${\dot{x}}^{B}=\;dx^{B}/\;dt$ are components of a tangent vector field.

The metric is denoted $g_{AB}\left(x\right)$. It is chosen to be the positive-definite solution of

(2) $$\partial_{C}g_{DB}=g_{BA}\Gamma_{CD}^{A},$$

if such a solution exists. This is the main assumption of the present work. The reason for this choice is explained below.

The connection coefficients $\Gamma_{BC}^{*A}$ of the dual-geometry, dual w.r.t. the metric tensor $g_{AB}$, are by definition fixed by

$$\partial_{C}g_{DB}=g_{BA}\Gamma_{CD}^{A}+g_{DA}\Gamma_{CB}^{*A}.$$

Assumption (2) is therefore equivalent to the assumption that a metric *g* exists so that the dual geometry has vanishing connection coefficients or, equivalently, that the coordinates $x^{A}$ are affine coordinates for the dual geometry.

A well-known theorem states that a geometry has vanishing curvature if and only if its dual has vanishing curvature. See, for instance, Theorem 3.3 of [10]. One concludes that the assumption that a metric g(x) exists for which (2) holds implies that the geometry has vanishing curvature because it is the dual of the Euclidean geometry.

The following result is what one expects intuitively.

**Proposition** **1.** *Let g be a solution of (2). The following are equivalent:*

*(a)*  *g is the Hessian of a potential Φ(x);*
*(b)*  *The connection with coefficients $\Gamma_{BC}^{A}$ is torsion-free.*

**Proof.**  (a) implies (b)

Use $g_{DB}=\partial_{D}\partial_{B}\Phi$ to rewrite (2) as

$$\partial_{C}\partial_{D}\partial_{B}\Phi =\left[\partial_{B}\partial_{A}\Phi \right]\Gamma_{CD}^{A}.$$

This implies either that $\Gamma_{CD}^{A}=\Gamma_{DC}^{A}$ or that $\partial_{B}\partial_{A}\Phi =0$ for all *x* in some region of $R^{5}$. The latter implies that the metric *g* is constant in that region and that the connection coefficients vanish. In both cases, the connection is torsion-free.

(b) implies (a)

If the connection is torsion-free, then (2) implies

$$g^{FB}\partial_{C}g_{DB}=\Gamma_{CD}^{F}=\Gamma_{DC}^{F}=g^{FB}\partial_{D}g_{CB}$$

with $g^{FB}$, the components of the inverse of *g*. This implies

$$\partial_{C}g_{DB}=\partial_{D}g_{CB}.$$

This expression is the necessary and sufficient condition in a simply-connected domain for the existence of functions $\eta_{B}$ such that

$$g_{DB}=\partial_{D}\eta_{B}.$$

Because the metric tensor is symmetric, it follows that $\partial_{D}\eta_{B}=\partial_{B}\eta_{D}$, which is the necessary and sufficient condition for the existence of a potential Φ(x) such that $\eta_{B}=\partial_{B}\Phi$. One concludes that

$$g_{DB}=\partial_{D}\eta_{B}=\partial_{D}\partial_{B}\Phi \left(x\right).$$

□

## 3. Inverse Formulas

From now on, raising and lowering indices is carried out using the 4-d metric tensor $g_{\mu \nu }$. In particular, $g^{\mu \rho }$ denotes the components of the inverse of the four-dimensional matrix with components $g_{\mu \nu }$. Note that the latter is positive-definite because the five-dimensional matrix *g* is positive-definite.

Introduce the notations

$$\begin{array}{cc} \; & \lambda^{\mu }=g^{\mu \rho }g_{\rho 4},\\ \; & \lambda_{\nu }=g_{\nu \mu }\lambda^{\mu }=g_{\nu 4}\\ \; & \gamma =g_{44}-\lambda_{\mu }g^{\mu \nu }g_{\nu 4}=g_{44}-\lambda_{\mu }\lambda^{\mu }. \end{array}$$

The vector field $\lambda_{\mu }$ controls the coupling of 4-d spacetime to the fifth dimension. Both $\lambda_{\mu }$ and the scalar field γ depend in principle on 5-d coordinates $x^{A}$.

**Proposition** **2.** *The positive-definitness of the metric tensor g implies that $0<\gamma \leq g_{44}$.*

**Proof.**  The scalar $\lambda_{\mu }\lambda^{\mu }$ is non-negative. This implies $\gamma \leq g_{44}$.

For any 5-vector, $u^{A}$ is

$$0\leq u^{A}g_{AB}u^{B}=u^{\mu }g_{\mu \nu }u^{\nu }+2u^{\mu }\lambda_{\mu }u^{4}+g_{44}{\left(u^{4}\right)}^{2},$$

with strict inequality if the vector *u* does not vanish. Choose $u^{\mu }=\lambda^{\mu }$ and $u^{4}=-1$. Then, it follows that 0<γ. □

The connection coefficients $\Gamma_{CD}^{A}$ can be calculated starting from the five-dimensional metric tensor *g*. This follows from

**Proposition** **3.** *Expression (2) implies*

(3) $$\begin{array}{cc} \gamma \Gamma_{CD}^{\rho }\; & =\gamma X^{\rho \sigma }\partial_{C}g_{D\sigma }-\lambda^{\rho }\partial_{C}g_{D4}, \end{array}$$

(4) $$\begin{array}{cc} \gamma \Gamma_{CD}^{4} & =\partial_{C}g_{D4}-\lambda^{\mu }\partial_{C}g_{D\mu } \end{array}$$

*with*

(5) $$X^{\rho \sigma }=g^{\rho \sigma }+\frac{1}{\gamma }\;\lambda^{\rho }\lambda^{\sigma }.$$

**Proof.**  Write (2) as

$$\begin{array}{cc} \partial_{C}g_{D\mu } & =g_{\mu \nu }\Gamma_{CD}^{\nu }+\lambda_{\mu }\Gamma_{CD}^{4},\\ \partial_{C}g_{D4} & =\lambda_{\nu }\Gamma_{CD}^{\nu }+g_{44}\Gamma_{CD}^{4}. \end{array}$$

Multiply the former line with $g^{\rho \mu }$ to obtain

(6) $$\Gamma_{CD}^{\rho }=g^{\rho \mu }\partial_{C}g_{D\mu }-\lambda^{\rho }\Gamma_{CD}^{4}.$$

Insert this result in the latter line. This gives

$$\partial_{C}g_{D4}=\lambda^{\mu }\partial_{C}g_{D\mu }+\gamma \Gamma_{CD}^{4}.$$

This is result (4). Combine (6) with (4) to obtain (3). □

Note that $\gamma X^{\rho \sigma }\lambda_{\sigma }=g_{44}\lambda^{\rho }$. The positive-definite tensor $X^{\rho \sigma }$ turns out to be a central object in what follows. The inverse of the tensor $X^{\nu \rho }$ is denoted $Y_{\mu \nu }$. It is given by

$$\begin{array}{cc} Y_{\mu \nu }\; & =\frac{1}{g_{44}}\left[g_{\mu \nu }g_{44}-\lambda_{\mu }\lambda_{\nu }\right]. \end{array}$$

It is positive-definite because it is the inverse of a positive-definite tensor.

## 4. Conserved Quantities

From now on, assume connection coefficients $\Gamma_{BC}^{A}$, which are such that a metric g(x) exists solving (2). Multiply expression (1) with $g_{DA}$ and use (2) to find

$$\begin{array}{cc} 0 & =g_{DA}{\ddot{x}}^{A}+g_{DA}\Gamma_{BC}^{A}{\dot{x}}^{B}{\dot{x}}^{C}\\ \; & =g_{DA}{\ddot{x}}^{A}+\left[\partial_{B}g_{CD}\right]\;{\dot{x}}^{B}{\dot{x}}^{C}\\ \; & =g_{DA}{\ddot{x}}^{A}+{\dot{x}}^{C}\frac{\;d\;}{\;dt}g_{CD}\\ \; & =\frac{\;d\;}{\;dt}\left[g_{DA}{\dot{x}}^{A}\right]. \end{array}$$

This shows that the quantities $P_{D}=g_{DA}{\dot{x}}^{A}$ are constant along any geodesic. Write them as

(7) $$\begin{array}{cc} P_{\mu } & =p_{\mu }+\lambda_{\mu }{\dot{x}}^{4}\;\mathrm{with}\;p_{\mu }=g_{\mu \nu }{\dot{x}}^{\nu }, \end{array}$$

(8) $$\begin{array}{cc} P_{4} & =\lambda_{\mu }{\dot{x}}^{\mu }+g_{44}{\dot{x}}^{4}. \end{array}$$

Solving these equations yields

(9) $$\begin{array}{cc} \gamma {\dot{x}}^{\nu } & =\gamma X^{\nu \mu }\;P_{\mu }-\lambda^{\nu }P_{4}, \end{array}$$

(10) $$\begin{array}{cc} \gamma {\dot{x}}^{4} & =P_{4}-\lambda^{\mu }P_{\mu }. \end{array}$$

The conserved quantities $P_{A}$ can be used as local affine coordinates w.r.t. a reference point $x_{0}$. The exponential map determines a point $x_{1}$ in 5d spacetime by following a geodesic $t\mapsto x_{t}$ with initial velocities ${\dot{x}}^{A}$ starting from the reference point $x_{0}$ at t=0 up to $x_{1}$ at t=1. In this way, the geodesic characterized by the conserved quantities $P_{A}$ is associated with a point $x_{1}$ in 5d spacetime.

## 5. Geodesics in 4-d Spacetime

Distinct geodesics in five dimensions with common starting point *x* can have the same initial velocity ${\dot{x}}^{\mu }$ when projected on four-dimensional spacetime, i.e., when neglecting what happens in the fifth dimension. They are characterized by the proposition following below. It shows that fixing the value of $P_{4}$ restores the one-to-one relation between initial velocity ${\dot{x}}^{\nu }$ and geodesic.

**Proposition** **4.** *Geodesics in five-dimensional space with the common starting point $x_{0}$ have the same initial 4-d velocity components ${\dot{x}}_{0}^{\mu }$ if and only if the conserved quantities $P_{A}$ that characterize the geodesic give the same value at the point $x_{0}$ to the quantities $g_{44}P_{\rho }-\lambda_{\rho }P_{4}$.*

**Proof.**  From (7) and (8), one obtains

$$g_{44}P_{\rho }-\lambda_{\rho }P_{4}=g_{44}p_{\rho }-\lambda_{\rho }\lambda_{\sigma }{\dot{x}}^{\sigma }=g_{44}Y_{\rho \sigma }{\dot{x}}^{\sigma }.$$

The r.h.s. of this expression depends only on the 5-d position *x* and on the 4-velocity ${\dot{x}}^{\mu }$ but not on ${\dot{x}}^{4}$. Hence, if the 4-velocities coincide at $x_{0}$ then the quantities $g_{44}P_{\rho }-\lambda_{\rho }P_{4}$ do concide as well.

The argument also works in the other direction because $g_{44}$ is strictly positive and the tensor $Y_{\rho \sigma }$ is invertible. □

Note that the starting point *x* of any geodesic is a point in 5-d. The restriction of the geodesic to four dimensions may still depend on the initial value of the fifth component $x^{4}$.

## 6. The Choice *P*_4_ = 0

From now on, the choice $P_{4}=0$ is made. In addition, a reference point $x_{0}$ is chosen in 5-d hyperspace. Then, by Proposition 4 there is a one-to-one correspondence between vectors $\dot{x}$ in the tangent space at $x_{0}$ and the geodesics labeled by the conserved quantities $P_{\mu }$. These geodesics define a geometry in the neighborhood of $x_{0}$. It is shown below that this geometry can be described by an effective connection not referring to the fifth dimension.

The choice $P_{4}=0$ implies that the 4-momenta $p_{\mu }$ are given by

$$p_{\mu }=\frac{g_{44}}{\gamma }P_{\mu }.$$

The spacetime positions along the geodesic characterized by the conserved quantities $P_{\mu }$ satisfy

(11) $${\dot{x}}^{\mu }=X^{\mu \nu }P_{\nu }.$$

The position $x^{4}$ in the fifth dimension satisfies

$${\dot{x}}^{4}=-\frac{1}{\gamma }\lambda^{\mu }P_{\mu }.$$

**Proposition** **5.** *Torsion-free effective connection coefficients ${\bar{\Gamma }}_{\mu \nu }^{\tau }$ exist such that any geodesic in 5-d satisfying $P_{4}=0$ also satisfies the 4-d geodesic equations*

(12) $${\ddot{x}}^{\tau }+{\bar{\Gamma }}_{\mu \nu }^{\tau }{\dot{x}}^{\mu }{\dot{x}}^{\nu }=0.$$

*The effective connection coefficients are given by*

(13) $$\begin{array}{cc} {\bar{\Gamma }}_{\mu \nu }^{\tau } & =\frac{1}{2}\Gamma_{\mu \nu }^{\tau }+\frac{1}{2{\left(g_{44}\right)}^{2}}\Gamma_{44}^{\tau }\lambda_{\mu }\lambda_{\nu }-\frac{1}{2g_{44}}\left(\Gamma_{\mu 4}^{\tau }+\Gamma_{4\mu }^{\tau }\right)\lambda_{\nu }\\ \; & +(\mu ↔\nu ). \end{array}$$

*Here, μ↔ν means that the preceeding expression is repeated with the symbols μ and ν interchanged.*

**Proof.**  Subtract (1) from (12) to obtain

(14) $$\begin{array}{cc} {\bar{\Gamma }}_{\mu \nu }^{\tau }\;{\dot{x}}^{\mu }{\dot{x}}^{\nu } & =\Gamma_{\mu \nu }^{\tau }\;{\dot{x}}^{\mu }{\dot{x}}^{\nu }+\Gamma_{44}^{\tau }{\left({\dot{x}}^{4}\right)}^{2}+\left(\Gamma_{\mu 4}^{\tau }+\Gamma_{4\mu }^{\tau }\right){\dot{x}}^{\mu }{\dot{x}}^{4}. \end{array}$$

Use that $P_{4}=0$ to obtain

$$\begin{array}{cc} \; & \gamma^{2}\left({\bar{\Gamma }}_{\mu \nu }^{\tau }-\Gamma_{\mu \nu }^{\tau }\right)X^{\mu \rho }P_{\rho }X^{\nu \sigma }P_{\sigma }\\ \; & =\Gamma_{44}^{\tau }{\left(\lambda^{\mu }P_{\mu }\right)}^{2}-\gamma \left(\Gamma_{\mu 4}^{\tau }+\Gamma_{4\mu }^{\tau }\right)X^{\mu \rho }P_{\rho }\lambda^{\sigma }P_{\sigma }. \end{array}$$

This condition should hold for all $P_{\mu }$. This implies

$$\begin{array}{cc} \; & \gamma^{2}\left({\bar{\Gamma }}_{\mu \nu }^{\tau }-\frac{1}{2}\Gamma_{\mu \nu }^{\tau }-\frac{1}{2}\Gamma_{\nu \mu }^{\tau }\right)X^{\mu \rho }X^{\nu \sigma }\\ \; & =\Gamma_{44}^{\tau }\lambda^{\rho }\lambda^{\sigma }-\frac{1}{2}\gamma \left(\Gamma_{\mu 4}^{\tau }+\Gamma_{4\mu }^{\tau }\right)X^{\mu \rho }\lambda^{\sigma }-\frac{1}{2}\gamma \left(\Gamma_{\mu 4}^{\tau }+\Gamma_{4\mu }^{\tau }\right)X^{\mu \sigma }\lambda^{\rho }. \end{array}$$

Multiply the above expression with $Y_{\rho \pi }Y_{\sigma \xi }$. This gives

$$\begin{array}{cc} \; & \gamma^{2}\left({\bar{\Gamma }}_{\pi \xi }^{\tau }-\frac{1}{2}\Gamma_{\pi \xi }^{\tau }-\frac{1}{2}\Gamma_{\xi \pi }^{\tau }\right)X^{\nu \sigma }\\ \; & =\Gamma_{44}^{\tau }Y_{\rho \pi }\lambda^{\rho }\;Y_{\sigma \xi }\lambda^{\sigma }-\frac{1}{2}\gamma \left(\Gamma_{\pi 4}^{\tau }+\Gamma_{4\pi }^{\tau }\right)Y_{\sigma \xi }\lambda^{\sigma }-\frac{1}{2}\gamma \left(\Gamma_{\xi 4}^{\tau }+\Gamma_{4\xi }^{\tau }\right)Y_{\rho \pi }\lambda^{\rho }. \end{array}$$

Finally use that

$$\lambda^{\sigma }Y_{\sigma \xi }=\frac{\gamma }{g_{44}}\lambda_{\xi }$$

to obtain (13). □

## 7. Parallel Transport

In the present section, the connection coefficients ${\bar{\Gamma }}_{\nu \rho }^{\tau }$ are those defined by (13). In particular, all geodesics under consideration satisfy $P_{4}=0$. An explicit expression is obtained for the parallel transport operators.

Choose basis vectors $e_{\mu }$ defined by $e_{\mu }^{\rho }=g_{\mu }^{\rho }$. The tangent vector $\dot{x}$ is then given by

$$\dot{x}={\dot{x}}^{\mu }\;e_{\mu }.$$

Parallel transport Π(t) along a smooth curve $t\mapsto x_{t}$ is represented by a matrix $\Pi_{\nu }^{\mu }\left(t\right)$ defined by

$$\Pi \left(t\right)e_{\nu }\left(0\right)=\Pi_{\nu }^{\mu }\left(t\right)\;e_{\mu }\left(t\right).$$

For convenience, here and at many occasions in what follows, *t* is written instead of $x_{t}$ and 0 instead of $x_{0}$. The operator Π(t) maps the tangent space at $x_{0}$ onto the tangent space at $x_{t}$. Choose

$$\Pi_{\nu }^{\mu }\left(t\right)=X^{\mu \rho }\left(t\right)Y_{\rho \nu }\left(0\right).$$

This choice of parallel transport determines a connection [13]. Let us show that it reproduces the geometry determined by the effective connection coefficients ${\bar{\Gamma }}_{\nu \rho }^{\tau }$. If $t\mapsto x_{t}$ is a geodesic determined by the conserved quantities $P_{\mu }$, as given by (11), then one has

(15) $$\begin{array}{cc} \Pi \left(t\right){\dot{x}}_{0} & ={\dot{x}}_{0}^{\nu }\;\Pi \left(t\right)e_{\nu }\left(0\right)\\ \; & ={\dot{x}}_{0}^{\nu }\;\Pi_{\nu }^{\mu }\left(t\right)\;e_{\mu }\left(t\right)\\ \; & =X^{\nu \sigma }\left(0\right)P_{\sigma }\;X^{\mu \rho }\left(t\right)Y_{\rho \nu }\left(0\right))e_{\mu }\left(t\right)\\ \; & =P_{\rho }\;X^{\mu \rho }\left(t\right)e_{\mu }\left(t\right)\\ \; & ={\dot{x}}^{\mu }\left(t\right)e_{\mu }\left(t\right)\\ \; & =\dot{x}\left(t\right). \end{array}$$

This shows that the operators Π(t) implement parallel transport for the connection studied in the previous sections.

## 8. The Pseudometric

Choose an arbitrary starting point *x* in spacetime and a pseudometric G(x) in the tangent plane at *x*. This pseudometric can be, for instance, that of Minkowski: G(x)=η with η=[+,−,−,−]. Next, use parallel transport to define the pseudometric *G* on a neighborhood of *x*. This gives

(16) $$G_{\mu \nu }\left(y\right)=Y_{\mu \tau }\left(y\right)X^{\tau \rho }\left(x\right)G_{\rho \sigma }\left(x\right)X^{\sigma \xi }\left(x\right)Y_{\xi \nu }\left(y\right).$$

**Proposition** **6.** *The parallel transport operators Π(x) defined in the previous section conserve the metric tensor $G_{\mu \nu }\left(y\right)$ given by (16).*

**Proof.**  A straightforward calculation shows that

$$\begin{array}{cc} {\left(\Pi \left(x\mapsto y\right)\;e_{\mu }\left(x\right),\Pi \left(x\mapsto y\right)e_{\nu }\left(x\right)\right)}_{y} & =G_{\mu \nu }\left(x\right)\\ \; & ={\left(e_{\mu },e_{\nu }\right)}_{x}. \end{array}$$

□

One concludes from this proposition that the geometry defined by the connection coefficients ${\bar{\Gamma }}_{\nu \rho }^{\tau }$ is the metric connection, also called the Levi–Civita connection, for the (pseudo)metric *G*.

An important property of the metric connection is that the square

$$|\dot{x}{|}^{2}={\dot{x}}^{\mu }{\bar{G}}_{\mu \nu }{\dot{x}}^{\nu }$$

of the length of the velocity 4-vector is constant along any geodesic $t\mapsto x_{t}$. If the square length vanishes, then the geodesic is called a null-geodesic.

Assume that *G* is given by (16). Then, one finds

(17) $$\begin{array}{cc} |\dot{x}{|}^{2} & ={\dot{x}}^{\mu }Y_{\mu \tau }\left(t\right)X^{\tau \rho }\left(0\right)G_{\rho \sigma }\left(0\right)X^{\sigma \xi }\left(0\right)Y_{\xi \nu }\left(t\right){\dot{x}}^{\nu }\\ \; & =P_{\tau }X^{\tau \rho }\left(0\right)G_{\rho \sigma }\left(0\right)X^{\sigma \xi }\left(0\right)P_{\xi }. \end{array}$$

If *v* is any vector of the null-cone at the reference point $x_{0}$, i.e., a vector satisfying $v^{\mu }G_{\mu \nu }\left(0\right)v^{\nu }$ = 0, then the geodesic with $P_{\mu }=Y_{\mu \nu }v^{\nu }$ starting at $x_{0}$ is a null-geodesic.

## 9. The de Sitter Space

The following example reproduces the geometry of the de Sitter space in the region |x|<L with *L* the de Sitter length and with

$$\left|x\right|={\left(\sum_{a=1}^{3}{\left(x^{a}\right)}^{2}\right)}^{1/2}.$$

Introduce a positive function α(x) defined by

$$\alpha =-1+\frac{L}{\sqrt{L^{2}-{\left|x\right|}^{2}}}.$$

Use it to define the metric tensor $g_{\mu \nu }$

(18) $$\begin{array}{cc} \; & g_{00}=g_{44}=1,\\ \; & g_{04}=\sqrt{\frac{\alpha }{1+\alpha }},\\ \; & g_{0a}=g_{a4}=0,\;a=1,2,3,\\ \; & g_{ab}=\delta_{ab}+\alpha \frac{x^{a}x^{b}}{{\left|x\right|}^{2}},\;a,b=1,2,3. \end{array}$$

It is easy to verify that the tensor *g* is positive-definite. In the limit of large *L*, the function α tends to zero. This implies that the metric tensor converges to the identity matrix.

From the definition of the function γ, one obtains

$$\gamma =\frac{1}{1+\alpha }.$$

The 4-by-4 tensor *Y* is found to be given by

(19) $$\begin{array}{cc} \; & Y_{00}=\frac{1}{1+\alpha },\\ \; & Y_{0a}=Y_{a0}=0,\;a=1,2,3\\ \; & Y_{a,b}=g_{ab},\;a,b=1,2,3. \end{array}$$

The inverse *X* of *Y* is the identity matrix at |x|=0. Hence, (16) with G(0)=η yields

(20) $$\begin{array}{cc} G_{\mu \nu }\left(y\right) & =Y_{\mu \tau }\left(y\right)\eta^{\tau \rho }Y_{\rho \nu }\left(y\right)\\ \; & =\left(\begin{array}{cc} {\left(1+\alpha \right)}^{2} & 0\\ 0 & -g^{2} \end{array}\right). \end{array}$$

This gives

(21) $$\begin{array}{cc} G_{00}\left(y\right) & =\frac{L^{2}-{\left|x\right|}^{2}}{L^{2}},\\ G_{0a}\left(y\right) & =0,\;a=1,2,3,\\ G_{ab}\left(y\right) & =-\delta_{ab}-\frac{x^{a}x^{b}}{L^{2}-{\left|x\right|}^{2}},\;a,b=1,2,3. \end{array}$$

This result is the pseudometric tensor of the de Sitter model in static coordinates [14].

## 10. Discussion

The geometry of spacetime is usually described by a pseudometric and the corresponding metric connection. For a free-falling observer, this pseudometric is in good approximation equal to the Minkowski metric. The present work considers a large class of models for which the connection is obtained from a curvature-free connection in a five-dimensional space by neglecting what happens in the fifth dimension. The curvature-free connection is the dual connection of the 5-d Euclidean geometry w.r.t. an arbitrary positive-definite metric. It is shown that the connection obtained in this way can be the metric connection for a well-chosen pseudometric.

The focus is on mathematical aspects. Physical relevance is not yet investigated. The embedding of spacetime in higher-dimensional spaces is an extensively studied topic. This raises the obvious question of how the present class of models relates to those of the existing literature. The viewpoint of neglecting what happens in the fifth dimension is close to that of induced matter theory, while the majority of studies adopt the point of view that spacetime is embedded in the higher-dimensional space as a curved hypersurface.

For the sake of simplicity, the dimension of the embedding space is taken equal to five. An extension of the present approach to higher dimensions is obvious. It would not alter the main conclusion of the work, namely, that it is meaningful to work with two metric tensors simultaneously, one of which is a Minkowski-like pseudometric and the other of which is positive-definite and is used to control the geometry.

A limitation of the present work is the assumption made in the final sections that the additional conserved quantity $P_{4}$ vanishes. The general case with $P_{4}\neq 0$ needs further investigation. The complication that arises is that effective forces induced by motion in the fifth dimension may be velocity-dependent.

## Data Availability

No additional data have been used.
